# 75% radiation dose reduction using deep learning reconstruction on low-dose chest CT

**DOI:** 10.1186/s12880-023-01081-8

**Published:** 2023-09-11

**Authors:** Gyeong Deok Jo, Chulkyun Ahn, Jung Hee Hong, Da Som Kim, Jongsoo Park, Hyungjin Kim, Jong Hyo Kim, Jin Mo Goo, Ju Gang Nam

**Affiliations:** 1 Department of Radiology, Seoul National University Hospital and College of Medicine, Seoul, 03080 Republic of Korea; 2 Department of Applied Bioengineering, Graduate School of Convergence Science and Technology, Seoul National University, Seoul, 08826 Republic of Korea; 3 ClariPi Research, Seoul, 03088 Republic of Korea; 4 Department of Radiology, Keimyung University Dongsan Hospital, Keimyung University School of Medicine, Daegu, 42601 Republic of Korea; 5 Department of Radiology, Busan Paik Hospital, College of Medicine, Inje University, Busan, 47392 Republic of Korea; 6 Department of Radiology, Yeungnam University Medical Center, Yeungnam University College of Medicine, Daegu, 42415 Republic of Korea; 7 Cancer Research Institute, Seoul National University, Seoul, 03080 Republic of Korea

**Keywords:** Artificial intelligence, Deep-learning image reconstruction, Noise reduction, Low-dose chest CT, Nodule detection

## Abstract

**Objective:**

Few studies have explored the clinical feasibility of using deep-learning reconstruction to reduce the radiation dose of CT. We aimed to compare the image quality and lung nodule detectability between chest CT using a quarter of the low dose (QLD) reconstructed with vendor-agnostic deep-learning image reconstruction (DLIR) and conventional low-dose (LD) CT reconstructed with iterative reconstruction (IR).

**Materials and methods:**

We retrospectively collected 100 patients (median age, 61 years [IQR, 53–70 years]) who received LDCT using a dual-source scanner, where total radiation was split into a 1:3 ratio. QLD CT was generated using a quarter dose and reconstructed with DLIR (QLD-DLIR), while LDCT images were generated using a full dose and reconstructed with IR (LD-IR). Three thoracic radiologists reviewed subjective noise, spatial resolution, and overall image quality, and image noise was measured in five areas. The radiologists were also asked to detect all Lung-RADS category 3 or 4 nodules, and their performance was evaluated using area under the jackknife free-response receiver operating characteristic curve (AUFROC).

**Results:**

The median effective dose was 0.16 (IQR, 0.14–0.18) mSv for QLD CT and 0.65 (IQR, 0.57–0.71) mSv for LDCT. The radiologists’ evaluations showed no significant differences in subjective noise (QLD-DLIR vs. LD-IR, lung-window setting; 3.23 ± 0.19 vs. 3.27 ± 0.22; *P* = .11), spatial resolution (3.14 ± 0.28 vs. 3.16 ± 0.27; *P* = .12), and overall image quality (3.14 ± 0.21 vs. 3.17 ± 0.17; *P* = .15). QLD-DLIR demonstrated lower measured noise than LD-IR in most areas (*P* < .001 for all). No significant difference was found between QLD-DLIR and LD-IR for the sensitivity (76.4% vs. 72.2%; *P* = .35) or the AUFROCs (0.77 vs. 0.78; *P* = .68) in detecting Lung-RADS category 3 or 4 nodules. Under a noninferiority limit of -0.1, QLD-DLIR showed noninferior detection performance (95% CI for AUFROC difference, -0.04 to 0.06).

**Conclusion:**

QLD-DLIR images showed comparable image quality and noninferior nodule detectability relative to LD-IR images.

**Supplementary Information:**

The online version contains supplementary material available at 10.1186/s12880-023-01081-8.

## Introduction

Low-dose chest CT (LDCT) is widely used for the diagnosis and follow-up of various lung diseases. Specifically, lung cancer screening using LDCT has been confirmed to reduce lung cancer mortality in several large-scale, randomized trials [1–3]; therefore, increasing number of nations are implementing lung cancer screening programs and recommending annual LDCT screening for high-risk asymptomatic individuals [4–6]. However, the cumulative radiation dose could be a major concern given the increasing number of LDCT examinations. With the technical advances achieved by CT vendors and improvements in reconstruction techniques, the radiation dose required for acquiring reliable chest CT images has steadily decreased. In particular, iterative reconstruction (IR), which substantially reduces image noise by sequentially adjusting the estimated reconstructions and the measured projections, has become a standard reconstruction technique for most CT vendors [7, 8].

Deep learning has been widely applied for various indications of medical imaging, including lesion detection, classification, segmentation, and noise reduction [9–11]. Several deep learning–based noise reduction algorithms have been proposed and tested in LDCT, and these algorithms have been reported to reduce noise and improve image quality substantially [12, 13]. Jiang et al. recently reported that lung nodule detection performance on ultralow-dose chest CT improved when using deep-learning reconstruction compared with IR [14]. However, insufficient evidence exists regarding whether deep-learning reconstruction may reproduce image quality and lesion detectability using images acquired with a decreased radiation dose. In this study, we aimed to evaluate the image quality and lung nodule detectability of CT images generated using a quarter of the low dose (QLD) and reconstructed with a commercial vendor-agnostic deep-learning image reconstruction (DLIR) in comparison with those of standard LDCT images reconstructed with a dedicated IR algorithm.

## Materials and methods

### Patients and LDCT

**Fig. 1:**
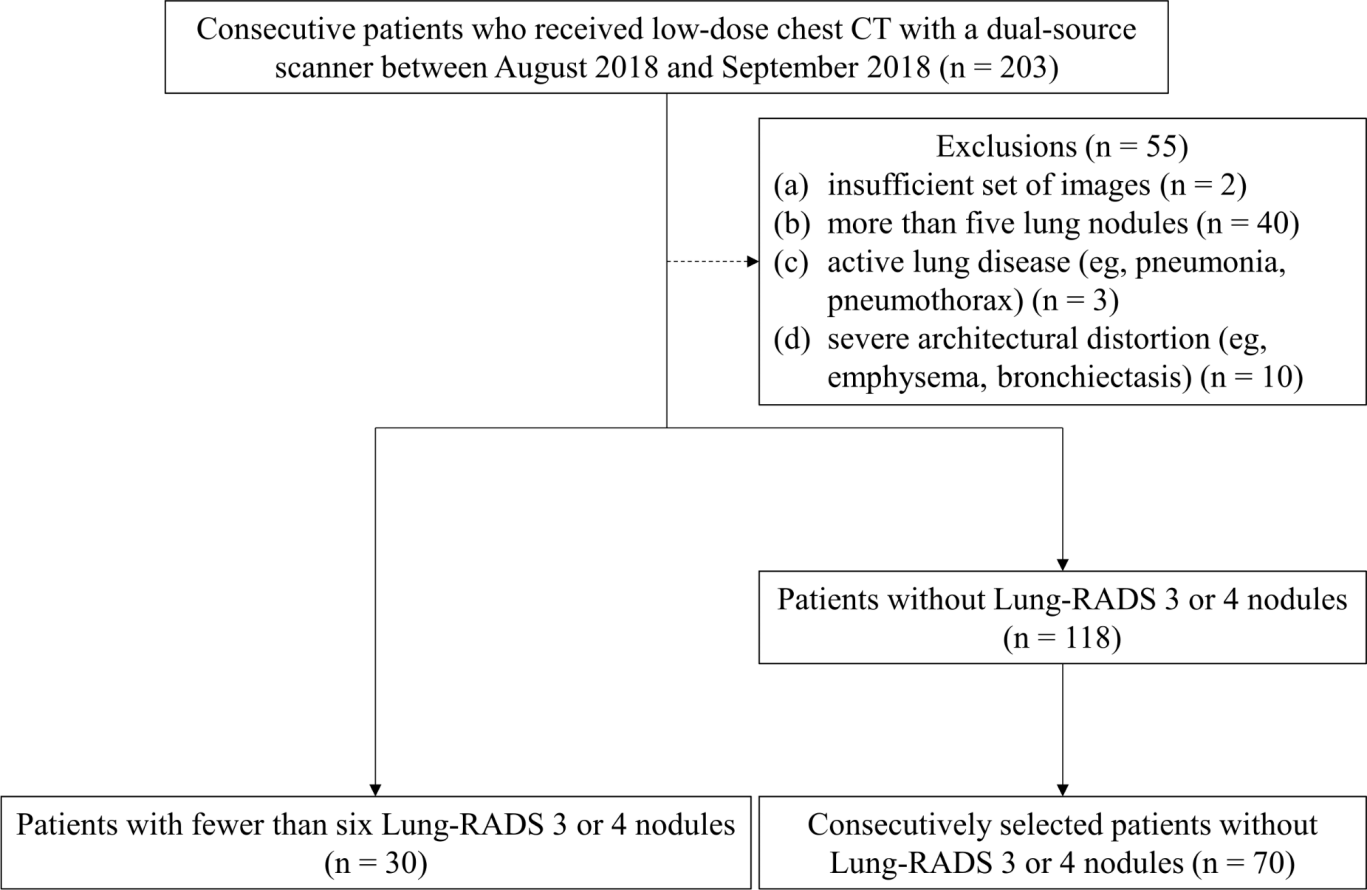
Patient selection flow diagram

Data from patients who underwent LDCT using a dual-source scanner (SOMATOM Force; Siemens Healthineers) between August 2018 and September 2018 were retrospectively collected at a tertiary care center (Seoul National University Hospital). All CT images were reviewed by a radiology resident (G.D.J. with 4 years of experience in chest CT interpretation). The patients were collected consecutively, while patients with more than five nodules, acute lung disease including pneumonia or pneumothorax, or severe architectural distortion were excluded to focus more clearly on the aims of the present study (Fig. 1). Demographic information (age, sex) and CT radiation dose information (CTDI_vol_, DLP, and effective dose) were documented. All CT images were reviewed and the presence of a lung nodule was determined consensually by a thoracic radiologist (J.G.N. with 9 years of experience) and a radiology resident (G.D.J.). The size of all nodules was calculated as the average of the maximal long-axis and the maximal perpendicular short-axis measurements.

### CT Acquisition and Image Reconstruction

For all LDCT scans, radiation was provided using two generators, whose radiation dose was split into a 1:3 ratio. QLD CT images were generated using the data acquired from a single generator, which provided a quarter dose of radiation, while standard-dose LDCT images were generated using the combined data from two generators. For each scan, the CT parameters were set as follows: tube voltage, 120 kVp; automatic tube current modulation by Care Dose 4D system (Siemens Healthineers) with quality reference tube-current-time product (15 mAs for one tube and 5 mAs for the other tube) and target CTDI_vol_ (1.36 mGy); detector collimation, 0.6 mm; detector pitch, 1.15; and gantry rotation period, 285 ms. The median scan range was 41.1 (IQR, 38.6–42.6) cm. Contrast media was not used.

**Fig. 2:**
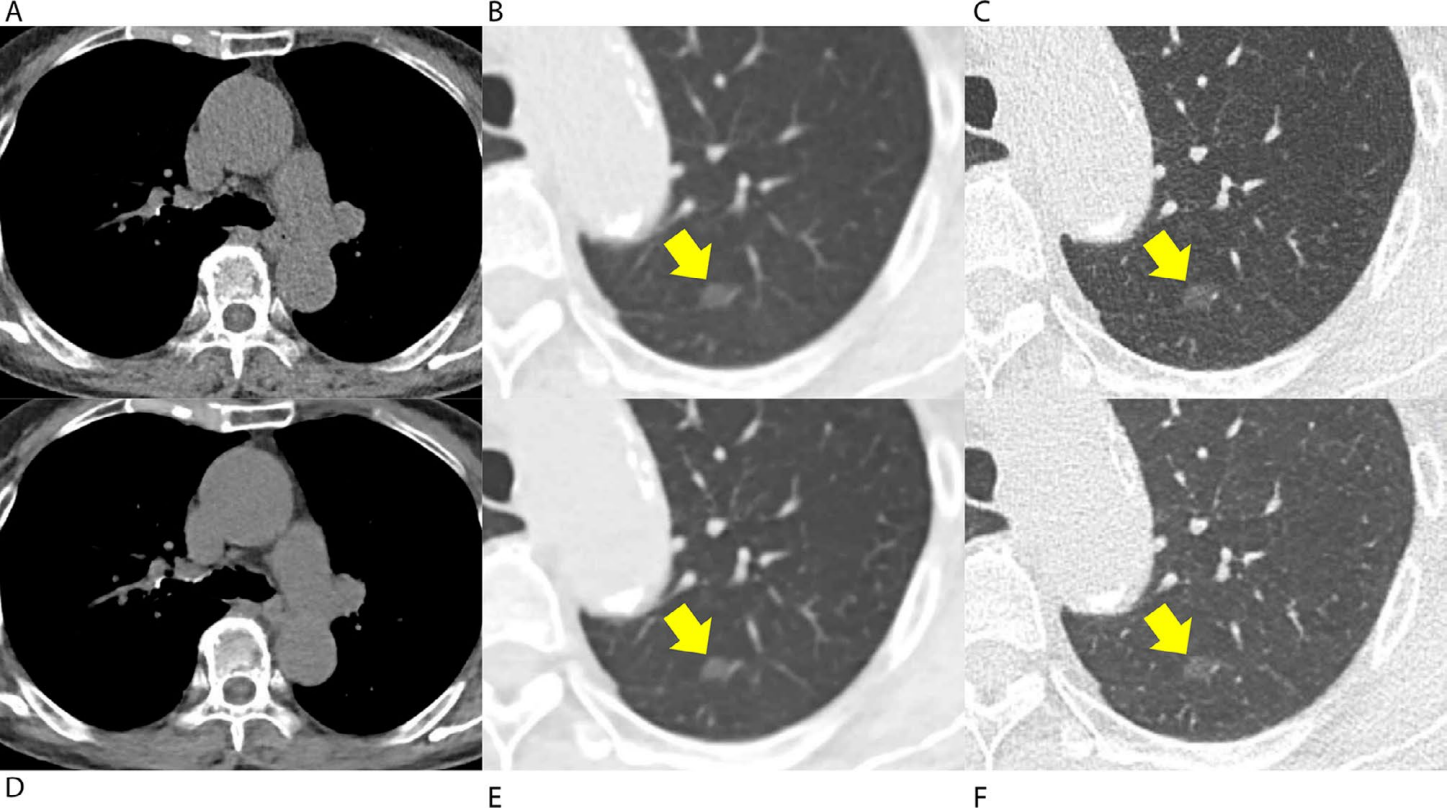
A woman received low-dose chest CT for the follow-up evaluation of a previously detected 8-mm ground-glass nodule in the left upper lobe. **(A-C)** Conventional low-dose chest CT images were reconstructed with iterative reconstruction, and **(D-F)** the images generated using a quarter dose of radiation were reconstructed with commercial deep-learning software. **(A, D)** Soft-tissue structures, including the aorta, subcutaneous fat, and paraspinal muscles, were visualized with a lower noise level in the mediastinal-window setting. **(C, F)** A ground-glass nodule in the left upper lobe was well visualized with a sharp margin on both images (arrow)

**Fig. 3:**
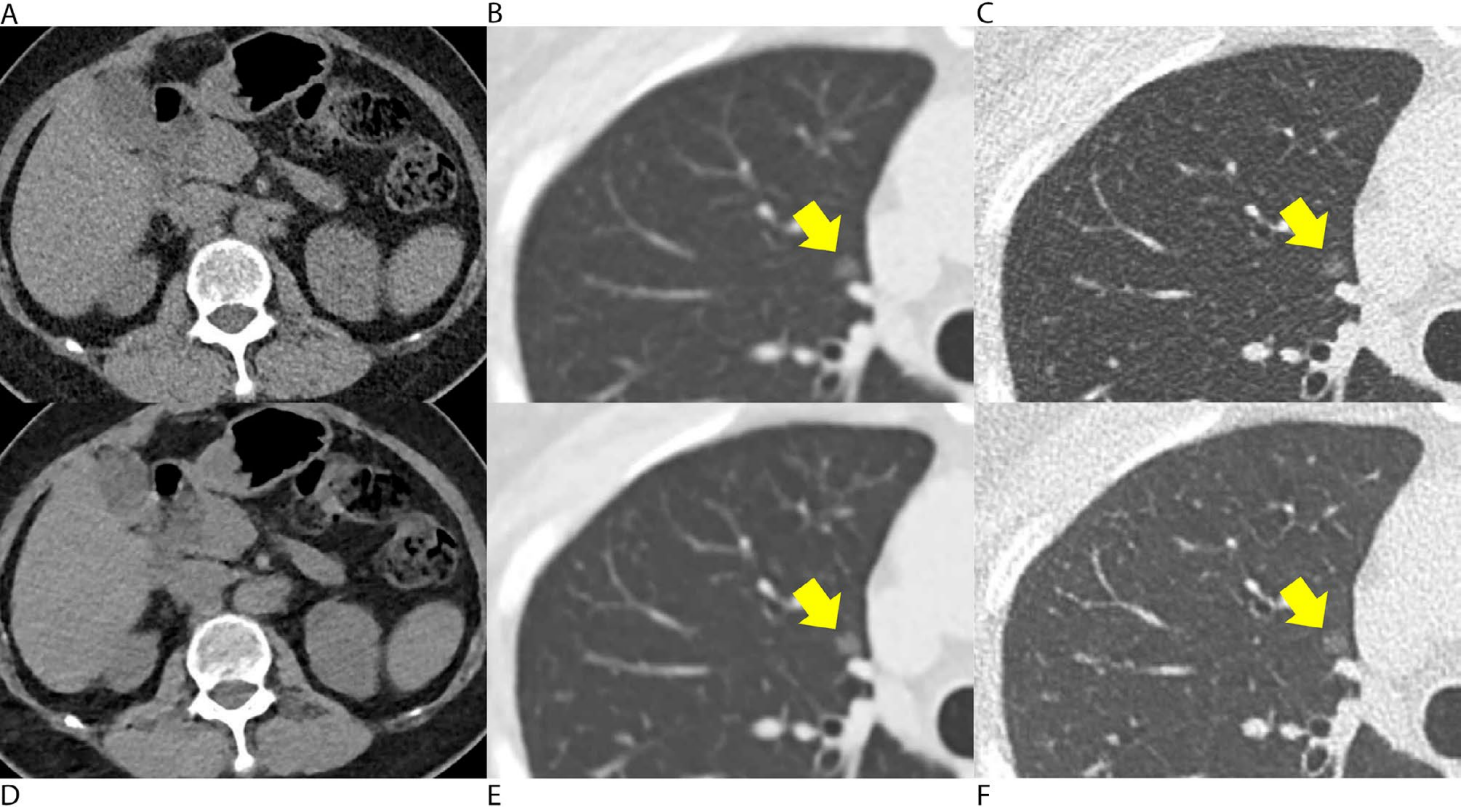
A woman received low-dose chest CT for the follow-up evaluation of multiple ground-glass nodules. **(A-C)** Conventional low-dose chest CT images were reconstructed with iterative reconstruction, and **(D-F)** the images generated using a quarter dose of radiation were reconstructed with commercial deep-learning software. **(A, D)** Soft-tissue structures, including the liver, spleen, kidney, and paraspinal muscles, were visualized with a lower noise level in the mediastinal-window setting on both images. **(C, F)** A ground-glass nodule in the right upper lobe was well visualized with a sharp margin on both images (arrow)

**Fig. 4:**
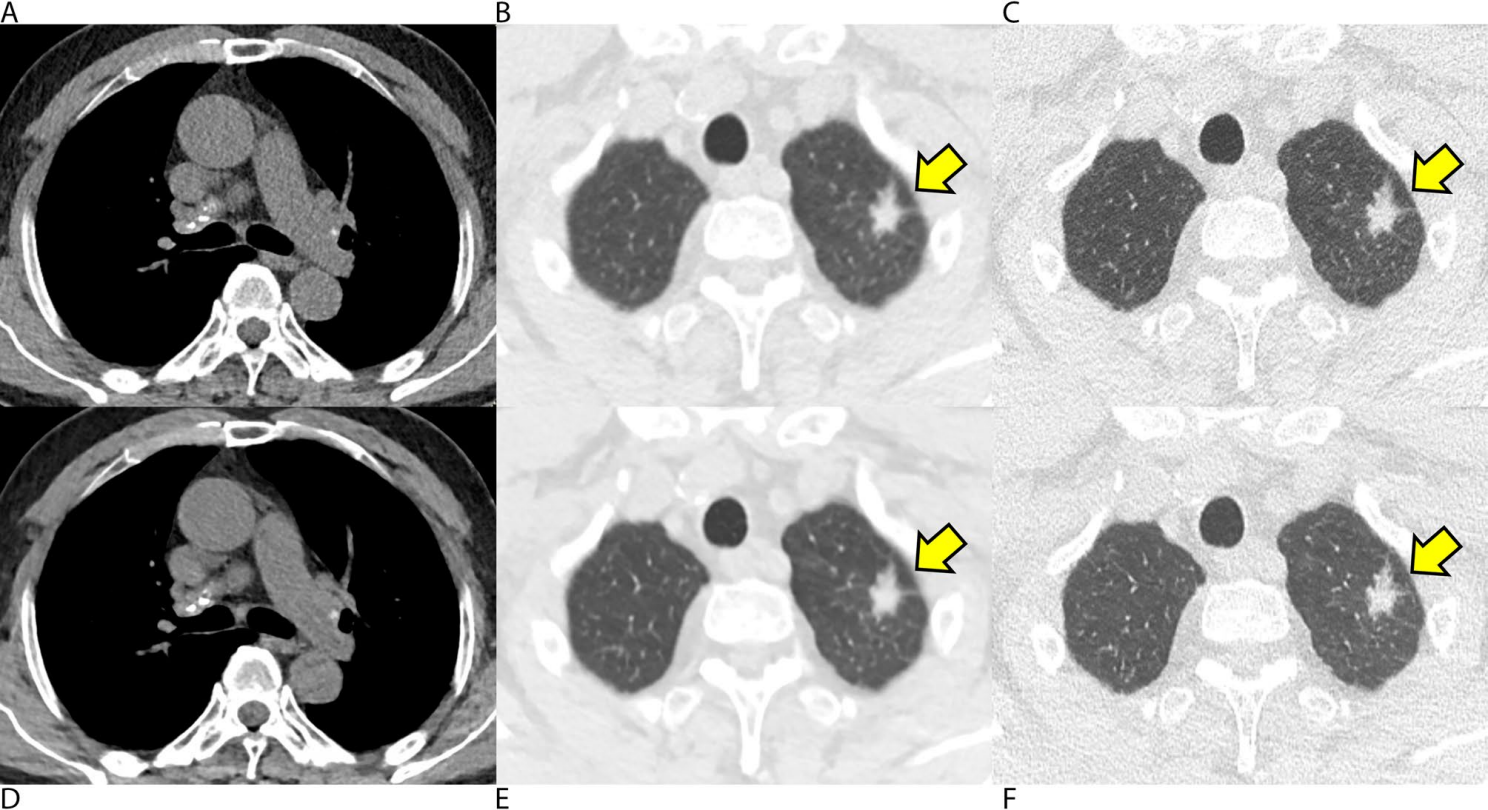
A man received low-dose chest CT for the follow-up evaluation of a spiculated lung nodule. **(A-C)** Conventional low-dose chest CT images were reconstructed with iterative reconstruction, and **(D-F)** the images generated using a quarter dose of radiation were reconstructed with commercial deep-learning software. **(A, D)** Soft-tissue structures including calcified mediastinal lymph nodes were visualized with a lower noise level in the mediastinal-window setting on both images. **(C, F)** A spiculated nodule in the left upper lobe was well visualized with a sharp margin on both images (arrow)

For image reconstruction, advanced modeled iterative reconstruction (ADMIRE, level 3) was applied to standard-dose LDCT images (hereafter, LD-IR images) [7], and deep-learning image reconstruction (high level for soft-kernel reconstruction, intermediate level for sharp-kernel reconstruction) was applied to QLD CT images (hereafter, QLD-DLIR images). Representative images are shown in Figs. 2, 3 and 4.

### Deep-learning image reconstruction

We used a commercial, vendor-agnostic deep-learning image reconstruction software (DLIR; ClariCT.AI, ClariPI Inc.), which has received European (CE Mark) and Korean regulatory approval (Korean Food and Drug Administration) [15]. This software takes filtered back-projection images as input and generates denoised images, and users can manipulate the optimal denoising level. This software is applicable to any type of filtered back projection images regardless of CT vendor, scan protocol, reconstruction kernel, and section thickness. A detailed description of the software is provided in Appendix E1 (online).

### Quantitative and qualitative image quality assessment

Image noise and the signal-to-noise ratio (SNR) were measured by a radiology resident (G.D.J.) in five different locations, including the lung parenchyma, trachea, aorta, muscle, and axillary fat (Figure E1). Image noise was defined as the standard deviation of the HU values within a region of interest larger than 0.5 cm^2^, while the signal-to-noise ratio was calculated as the absolute average HU value divided by the noise. To assess the spatial resolution, the edge-rise-distance (ERD) was measured semi-automatically at pulmonary vessels running in the axial plane. The ERD was defined as the distance between two points yielding 10% and 90% of the maximal intravascular HU values [12, 16]. All quantitative measures were evaluated for both 3-mm section-thickness standard-kernel images and 1-mm section-thickness sharp-kernel images.

For a qualitative assessment, three fellowship-trained thoracic radiologists (J.H.H., D.S.K., J. P. with 8–10 years of experience) evaluated the image quality of 200 randomly arranged image sets (QLD-DLIR and LD-IR) from 100 patients. Each radiologist assessed all 200 sets of images and these images were randomly distributed, ensuring that pairs of QLD-DLIR and LD-IR images from the same patient were not presented together. The radiologists independently reviewed subjective noise, spatial resolution, the presence of artifacts (distortion and beam-hardening artifacts), and overall image quality using a 4-point scale (1–4; a higher score indicated better image quality, Table E1 [online]). A distortion artifact was defined as the presence of image distortion generated from image reconstruction algorithms, typically false miliary nodules on the lung-window setting and granular distortion of mediastinal structures [12]. The primary evaluation was conducted using 3-mm section-thickness standard-kernel images, and 1-mm section-thickness sharp-kernel images were provided as a pair. The radiologists were blinded to patients’ demographics, clinical indications, and the reconstruction technique of the images. Inter-reader agreement was assessed using the intraclass correlation coefficient (ICC) based on a two-way mixed-effect model incorporating consistency and average measures. The agreement levels were categorized as follows: poor (< 0.50), fair (0.50–0.75), good (0.75–0.90), and excellent (0.90–1.00).

### Lung nodule detectability assessment

For nodule detectability, a performance test was conducted using the same 200 randomly arranged image sets used in the qualitative assessment. The three aforementioned fellowship-trained thoracic radiologists detected and localized all clinically significant nodules (solid or part-solid nodules ≥ 6 mm; Lung-RADS category 3 or 4) [17]. The radiologists rated their confidence in lesion detection using a 5-point scale, where a higher score indicated higher confidence in the presence of a Lung-RADS category 3 or 4 nodule [18]. For evaluation, 3-mm section-thickness standard-kernel images were provided as main images, and 1-mm section-thickness sharp-kernel images were provided as a pair for the further evaluation of nodule morphology (i.e., solid or subsolid) and to make accurate measurements. The radiologists were blinded to patients’ demographics, clinical indications, and the reconstruction technique of the images.

### Statistical analysis

Image quality metrics from QLD-DLIR and LD-IR were compared using the Wilcoxon signed-rank test or paired *t*-test, as appropriate. To assess lung nodule detectability, area under the jackknife free-response receiver operating characteristic curves (AUFROCs) were evaluated and compared between QLD-DLIR and LD-IR images, and the noninferiority limit was established as -0.1 [19, 20]. Sensitivity and specificity were compared using the McNemar test for individual radiologists and generalized estimating equations based on an exchangeable correlation matrix for the pooled radiologists. AUFROC analysis was performed using JAFROC version 4.2.1 and ICC was calculated using MedCalc version 20.218 (MedCalc software, Mariakerke, Belgium), while other statistical analyses were performed using SPSS version 25 (IBM Corp., Armonk, NY, USA). The statistical analyses were conducted by two radiologists (G.D.J. and J.G.N.) with 4 and 9 years of experience in medical statistical analyses. For all tests, *P* < .05 indicated statistical significance.

## Results

### Patient characteristics, clinical indications, nodule types, and radiation exposure

This study included 100 patients [median (IQR), 61 (53–70) years] including 52 men and 48 women (Table 1). LDCT was mostly indicated for the evaluation of thoracic metastasis (follow-up or initial workup; n = 57) or the follow-up of underlying thoracic diseases (n = 33; including lung nodules [n = 22], emphysema [n = 3], and others [n = 8]). Ten other patients received chest CT for a preoperative/pretransplantation workup (n = 2), further evaluation of abnormalities found on chest radiography (n = 7), or chronic cough (n = 1). Among the 100 patients, 30 had at least one clinically significant nodule (Lung-RADS category 3 or 4). In total, 48 Lung-RADS category 3 (n = 28) or 4 (n = 20) nodules were present; four were subsolid nodules and 44 were solid nodules. The median CTDI_vol_ and DLP were 0.29 (IQR, 0.25–0.32) mGy and 11.6 (IQR, 10.2–12.7) mGy*cm for QLD CT and 1.2 (IQR, 1.0–1.3) mGy and 46.4 (IQR, 41.0–50.8) mGy*cm for LDCT, respectively. The median effective dose was 0.16 (IQR, 0.14–0.18) mSv for QLD CT and 0.65 (IQR, 0.57–0.71) mSv for LDCT.

### Quantitative image quality assessment

The QLD-DLIR images showed lower noise and higher SNR than the LD-IR images in most evaluated areas (*P* < .001), except for trachea in 3-mm, standard-kernel images, for which the QLD-DLIR images showed higher noise (16.0 ± 3.9 vs. 13.4 ± 2.0; *P* < .001) and a lower SNR (64.3 ± 14.6 vs. 75.8 ± 11.7; *P* < .001) than the LD-IR images (Table 2). The ERD of the QLD-DLIR images was lower than that of the LD-IR images (1.52 ± 0.08 vs. 1.80 ± 0.11; *P* < .001) in 3-mm, standard-kernel images, indicating the superior spatial resolution of QLD-DLIR images. In 1-mm, sharp-kernel images, however, the ERD of QLD-DLIR images was higher than that of LD-IR images (1.15 ± 0.07 vs. 1.06 ± 0.08; *P* < .001).

### Qualitative image Quality Assessment

For 3-mm, standard-kernel images, the QLD-DLIR and LD-IR images did not show significant differences in overall image quality (QLD-DLIR vs. LD-IR; 3.14 ± 0.21 vs. 3.17 ± 0.17; *P* = .15). Specifically, the QLD-DLIR and LD-IR images showed similar quality in terms of subjective noise (QLD-DLIR vs. LD-IR; 3.23 ± 0.19 vs. 3.27 ± 0.22; *P* = .11), spatial resolution (3.14 ± 0.28 vs. 3.16 ± 0.27; *P =* .12), and distortion artifacts (3.08 ± 0.24 vs. 3.07 ± 0.19; *P* = .78; Table 3) in the lung-window setting; however, the QLD-DLIR images showed lower scores for subjective noise, spatial resolution, and distortion artifacts in the mediastinal-window setting, as well as the presence of beam-hardening artifacts (Table 3, Figure E2). For 1-mm, sharp-kernel images, the QLD-DLIR images showed lower image quality in all aspects, including overall image quality, when compared with LD-IR images (Table E2). The radiologists showed poor inter-reader agreement in assessing subjective noise (lung window setting) and presence of distortion artifact (Table 3).

### Nodule detectability assessment

Among the CT scans of the 100 included patients, 48 Lung-RADS category 3 (n = 28) or 4 (n = 20) nodules (4 subsolid and 44 solid nodules) were found in 30 patients, and those nodules were regarded as positive nodules, while Lung-RADS category 2 nodules were regarded as negative. In patient-based analyses, all three thoracic radiologists showed similar sensitivity and specificity values when using QLD-DLIR and LD-IR images (*P* > .05 for all, Table E3). The pooled radiologists showed sensitivity values of 78.9% (71/90) and 81.1% (73/90, *P* = .45) using QLD-DLIR and LD-IR images, respectively, and specificity values of 83.3% (175/210) and 84.3% (177/210; *P* = .74). On nodule-based analyses, all three thoracic radiologists showed similar nodule-based sensitivity (number of detected true-positive nodules divided by the total number of positive nodules) and false-positive rates (number of false-positive nodules divided by the total number of patients) using QLD-DLIR and LD-IR images (*P* > .05 for all; Table 4). In the pooled analysis, the nodule-based sensitivity values were 76.4% (110/144) and 72.2% (104/144; *P* = .35) when using QLD-DLIR and LD-IR, respectively, while the false-positive rates were 0.34 (102/300) and 0.34 (102/300; *P* = 1.00), each. In the JAFROC analysis, all three thoracic radiologists showed similar AUFROCs in detecting Lung-RADS 3 or 4 nodules using QLD-DLIR and LD-IR images (0.72 vs. 0.75. *P* = .38; 0.86 vs. 0.82, *P* = .36; and 0.72 vs. 0.75, *P* = .52). The pooled AUFROC also did not show a significant difference (0.77 vs. 0.78; *P* = .68) between the QLD-DLIR and LD-IR images. With a noninferiority limit of -0.1, the three thoracic radiologists showed noninferior detection performance using QLD-DLIR instead of LD-IR (95% CI for AUFROC difference; -0.04, 0.06). Representative cases for lung nodules are provided in Figs. 2, 3 and 4 and an example of a false-negative case is provided in Figure E3.

## Discussion

In this study, we compared the image quality of chest CT generated using a quarter dose of radiation and reconstructed with commercial deep-learning software (QLD-DLIR) to that of conventional LDCT images generated using full radiation dose and reconstructed with a dedicated IR technique (LD-IR). In the quantitative analysis, the QLD-DLIR images showed overall better noise, SNR, and ERD than the LD-IR images, suggesting better noise, image contrast, and spatial resolution, respectively. In the subjective, qualitative assessment, the QLD-DLIR and LD-IR images received comparable image quality scores in the lung evaluation, whereas the QLD-DLIR images showed lower spatial resolution with more noise and artifacts in the mediastinal evaluation. The three thoracic radiologists found no significant differences in overall image quality between the QLD-DLIR and LD-IR images. The detection performance of significant lung nodules was also evaluated. The radiologists did not show significantly different performance in detecting Lung-RADS 3 or 4 nodules on the QLD-DLIR and LD-IR images. The noninferiority of QLD-DLIR relative to LD-IR was confirmed.

The radiation dose required for reliable lung evaluation has substantially decreased since the introduction of IR, enabling LDCT to become the mainstream CT protocol in screening for lung diseases, including lung cancer. More recently, deep learning–based reconstruction has demonstrated excellent noise-reduction power, surpassing IR, suggesting the possibility of further radiation dose reduction [21–23]. Several studies have reported the feasibility of ultralow-dose chest CT images reconstructed with deep learning software [12, 14, 24]. However, those previous publications have not yet provided concrete evidence that deep learning may actually reduce the radiation dose required for reliable lung evaluations, as deep learning–reconstructed ultralow-dose CT scans have not been compared with conventional LDCT reconstructed using conventional IR. In this study, we attempted a 75% dose reduction in LDCT using commercial deep-learning reconstruction software. By using a dual-source scanner, we obtained quarter-dose and full-dose CT images simultaneously from a single scan, without imposing additional radiation.

The commercial deep-learning software we used in this study, ClariCT.AI, has the advantage of being applicable to any CT images (vendor-agnostic) without any vendor-specific adaptations. The software was trained with multi-vendor images through the synthetic sinogram-based low-dose simulation technique [25], producing generalizable denoising quality for diverse images. It has also been reported to produce less deep learning–specific image distortion [12], possibly by preserving the noise frequency spectrum during the denoising process. We tested a single software instead of various deep-learning reconstruction models, as it was technically the only applicable commercial deep-learning denoising software for the CT images taken from our dual-source scanner; however, a further comparison with other deep-learning models using multi-vendor CT scans would be warranted.

To assess the clinical feasibility of the images, we compared nodule detectability between QLD-DLIR and LD-IR. Detecting lung nodules is one of the major indications of CT, especially in the screening setting. We considered Lung-RADS category 3 or 4 nodules as positive, since those nodules alter follow-up plans in lung cancer screening programs [17]. Three thoracic radiologists showed no significant differences in sensitivity, specificity, false-positive rates, and AUFROC between QLD-DLIR and LD-IR. When nodules missed by the radiologists were reviewed (false-negative), the nodules were visualized comparably in both LD-IR and QLD-DLIR images (Figure E3), suggesting that the missing of nodules was mainly attributed to random human error rather than a different imaging technique. Noninferior nodule detection performance of QLD-DLIR over LD-IR was demonstrated in the JAFROC analysis. Jiang et al. also demonstrated the feasibility of deep learning–reconstructed ultralow-dose CT for nodule detection [14]; however, that study did not compare the performance to conventional LDCT images. Our study results suggest that radiation dose reduction up to 75% could be tried for LDCT scans conducted for the purpose of lung nodule screening. However, further validation on diverse vendors in detecting diverse abnormalities other than lung nodules should be warranted.

While the QLD-DLIR images exhibited superior results over the LD-IR images for most parameters in the quantitative image quality assessment, the radiologists assessed that LD-IR showed comparable to better image quality than QLD-DLIR for most parameters. Of particular note, the radiologists gave LD-IR better scores in overall image quality for 1-mm, sharp-kernel images. This discrepancy may primarily be due to the following two reasons. First, the radiologists were more accustomed to the texture of IR-reconstructed images and were relatively unfamiliar with DLIR-reconstructed images. In addition, the radiologists found a considerably higher level of beam-hardening artifacts (3.01 vs. 3.47; *P* < .001), which was not assessed by the quantitative measures, and this might have affected the overall image quality assessment. Second, as DLIR was trained to reduce quantitative metrics, typically measured image noise, DLIR might have advantages in quantitative assessment. As we optimized the DLIR settings mainly for 3-mm, standard-kernel images, the 1-mm, sharp-kernel images generally yielded lower scores. Further optimization of DLIR for each image type and the addition of a beam-hardening artifact–reduction algorithm would enhance image quality and reader preferences for QLD-DLIR images.

Our study has some limitations. First, because of its retrospective nature, selection bias could have affected the comparison of image quality and lung nodule detection performance. To minimize selection bias, patients were selected consecutively. In addition, patients with six or more nodules were excluded to focus the review on nodule detection performance, which could have also yielded additional selection bias. Second, images were taken using a single CT scanner, limiting the generalizability of the study results. Third, only one deep-learning reconstruction software was tested. Fourth, the number of subsolid nodules included in this study was small (n = 4), and thus meaningful subgroup analysis for subsolid nodules was limited. Further studies assessing if DLIR may properly preserve the morphology and size of the subsolid nodules would be beneficial. Fifth, the radiologists showed poor inter-reader agreement in assessing some qualitative parameters including subjective noise and presence of distortion artifacts. In addition, the radiologists gave comparable to lower scores to QLD-DLIR in assessing subjective noise while it showed lower measured noise level, possibly affected by different image textures or variable reader-familiarity to the technique. Lastly, the limit for noninferiority in nodule detection performance was set empirically rather than from preliminary analyses of nodule detection performance.

In conclusion, deep learning–reconstructed QLD images showed comparable image quality and noninferior nodule detectability to standard LDCT images reconstructed with IR.

**Table 1:** Patient Characteristics and Radiologic Findings

Variable	Number
**Demographics**	
Age (median [IQR])	61 [53–70]
Sex	
Men	52 (52%)
Women	48 (48%)
**CT information**	
Indication for CT	
Metastasis evaluation	57 (57%)
Follow-up of underlying diseases	33 (33%)
Preoperative/pretransplantation workup	2 (2%)
Chest radiograph abnormality	7 (7%)
Chronic cough	1 (1%)
**CTDI**_**vol**_ **(mGy; median [IQR])**	
LDCT	1.2 [1.0–1.3]
QLD CT	0.29 [0.25–0.32]
DLP (mGy*cm; median [IQR])	
LDCT	46.4 [41.0–50.8]
QLD CT	11.6 [10.2–12.7]
**Effective dose (mSv; median [IQR])**	
LDCT	0.65 [0.57–0.71]
QLD CT	0.16 [0.14–0.18]
Patients with Lung-RADS category 3 or 4 nodules	30 (30%)
**Number of lung nodules**	48
Lung-RADS category 3	28 (58%)
Lung-RADS category 4 A	16 (33%)
Lung-RADS category 4B	4 (8%)
**Radiologic nodule type**	
Subsolid	4 (8%)
Solid	44 (92%)

Note.—Categorical variables are presented as counts (%) and continuous variables as median [IQR].

CTDI_vol_=volume CT dose index, LDCT = low-dose CT, IQR = interquartile range, Lung-RADS = lung imaging reporting and data system, QLD = quarter of the low dose.

**Table 2:** Quantitative Image Quality Assessment Results

	QLD-DLIR, (3-mm thickness, standard kernel)	LD-IR (3-mm thickness, standard kernel)	*P*-value*	QLD-DLIR (1-mm thickness, sharp kernel)	LD-IR (1-mm thickness, sharp kernel)	*P*-value*
**Noise**						
Lung	*11.5 ± 3.3*	16.0 ± 3.8	< 0.001	*37.1 ± 6.7*	66.5 ± 10.9	< 0.001
Trachea	16.0 ± 3.9	*13.4 ± 2.0*	< 0.001	*44.2 ± 7.7*	57.5 ± 8.4	< 0.001
Aorta	*8.6 ± 2.3*	17.6 ± 2.2	< 0.001	*81.8 ± 6.1*	104.9 ± 7.2	< 0.001
Muscle	*12.2 ± 3.3*	22.8 ± 3.3	< 0.001	*81.0 ± 9.5*	111.8 ± 9.9	< 0.001
Axillary fat	*9.2 ± 2.0*	17.4 ± 3.3	< 0.001	*82.1 ± 11.6*	108.3 ± 16.6	< 0.001
**SNR**						
Lung	*84.4 ± 24.3*	58.9 ± 14.1	< 0.001	*23.0 ± 4.6*	13.7 ± 2.5	< 0.001
Trachea	64.3 ± 14.6	*75.8 ± 11.7*	< 0.001	*20.1 ± 3.6*	16.6 ± 2.7	< 0.001
Aorta	*5.6 ± 1.3*	2.6 ± 0.4	< 0.001	*0.58 ± 0.06*	0.43 ± 0.06	< 0.001
Muscle	*5.0 ± 1.4*	2.5 ± 0.5	< 0.001	*0.71 ± 0.12*	0.49 ± 0.08	< 0.001
Axillary fat	*11.3 ± 2.8*	6.0 ± 1.2	< 0.001	*1.2 ± 0.2*	0.95 ± 0.16	< 0.001
**Edge-rise-distance (mm)**	*1.52 ± 0.08*	1.80 ± 0.11	< 0.001	1.15 ± 0.07	*1.06 ± 0.08*	< 0.001

Note.—Data are presented as means ± standard deviations. Italicized data indicate that the values are lower (for noise and edge-rise-distance) or higher (for SNR) than the compared counterpart. DLIR = deep-learning image reconstruction, IR = iterative reconstruction, LD = low dose, QLD = quarter of the low dose, SNR = signal-to-noise ratio

*P-values were calculated using the paired *t*-test

**Table 3:** Qualitative Image Quality Assessment Results

	QLD-DLIR	LD-IR	*P*-value*	ICC [95% CI]
**Lung-window setting**				
Subjective noise	3.23 ± 0.19 (3, 3.33)	3.27 ± 0.22 (3, 3.33)	0.11	0.26 [0.12, 0.38]
Spatial resolution	3.14 ± 0.28 (3, 3.33)	3.16 ± 0.27 (3, 3.33)	0.12	0.51 [0.42, 0.59]
Distortion artifact	3.08 ± 0.24 (3, 3.33)	3.07 ± 0.19 (3, 3)	0.78	0.52 [0.43, 0.60]
**Mediastinal-window setting**				
Subjective noise	3.00 ± 0.19 (3, 3)	*3.05 ± 0.14* *(3, 3)*	0.045	0.88 [0.85, 0.87]
Spatial resolution	2.92 ± 0.29 (2.67, 3)	*3.22 ± 0.29* *(3, 3.33)*	< 0.001	0.39 [0.28, 0.49]
Distortion artifact	2.75 ± 0.18 (2.67, 2.92)	*3.04 ± 0.14* *(3, 3)*	< 0.001	-0.34 [-0.58, -0.13]
**Beam-hardening artifact**	3.01 ± 0.35 (2.75, 3.33)	*3.47 ± 0.27* *(3.33, 3.67)*	< 0.001	0.64 [0.57, 0.69]
**Overall image quality**	3.14 ± 0.21 (3, 3.33)	3.17 ± 0.17 (3, 3.33)	0.15	0.45 [0.35, 0.54]

Note.—Results from 3-mm, standard-kernel images are presented. Data are presented as means ± standard deviations (interquartile range). Higher scores indicate better image quality. Italicized data indicate that the values are higher than the compared counterpart. CI = confidence interval, DLIR = deep-learning image reconstruction, ICC = intraclass correlation coefficient, IR = iterative reconstruction, LD = low dose, QLD = quarter of the low dose

*P-values were calculated using the paired *t*-test or Wilcoxon signed-rank test, as appropriate

**Table 4:** Nodule-Based Estimates of the Detection of Lung-RADS Category 3 or 4 Nodules

	AUFROC			Sensitivity			FP rate		
	QLD-DLIR	LD-IR	*P*-value*	QLD-DLIR	LD-IR	*P*-value*	QLD-DLIR	LD-IR	*P*-value*
Reader 1	0.72 (0.62–0.82)	0.75 (0.66–0.85)	0.38	77.1% (37/48)	72.9% (35/48)	0.73	0.29 (29/100)	0.36 (36/100)	0.29
Reader 2	0.86 (0.78–0.94)	0.82 (0.74–0.91)	0.36	79.2% (38/48)	68.8% (33/48)	0.30	0.10 (10/100)	0.10 (10/100)	1.00
Reader 3	0.72 (0.63–0.82)	0.75 (0.66–0.84)	0.52	72.9% (35/48)	75.0% (36/48)	1.00	0.63 (63/100)	0.56 (56/100)	0.31
Pooled readers	0.77 (0.70–0.83)	0.78 (0.71–0.85)	0.68	76.4% (110/144)	72.2% (104/144)	0.35	0.34 (102/300)	0.34 (102/300)	1.00

Note.—AUFROC values are presented with 95% confidence intervals. FP rates were calculated as the total number of FP nodules divided by the total number of patients (n = 100)

AUFROC = area under the jackknife free-response receiver operating characteristic curve, DLIR = deep-learning image reconstruction, FP = false positive, IR = iterative reconstruction, LD = low dose, Lung-RADS = lung imaging reporting and data system, QLD = quarter of the low dose

*P-values were calculated using jackknife free-response receiver operating characteristic curve analysis (for the AUFROC), the McNemar test (for the sensitivity of individual radiologists), the chi-square test (for the FP rate of individual radiologists), or generalized estimating equations (for the sensitivity and specificity of pooled radiologists)

### Electronic supplementary material

Below is the link to the electronic supplementary material.

Supplementary Material 1

## Data Availability

The datasets generated or analyzed during the study are available from the corresponding author on reasonable request.
