# Supplementary material for: 75% radiation dose reduction using deep learning reconstruction on low-dose chest CT
Source: BMC Med Imaging. 2023 Sep 11;23:121. doi: 10.1186/s12880-023-01081-8 (PMC10494344; doi:10.1186/s12880-023-01081-8)
Supplement: Supplementary file 1 — Supplementary Material 1 [file 12880_2023_1081_MOESM1_ESM.docx]

**SUPPLEMENTAL TEXT**

**Appendix E1**

**Commercial Deep-Learning Image Reconstruction Software**

The deep-learning image reconstruction (DLIR; ClariCT.AI, ClariPI Inc.) used in this study was developed to produce lower-noise images from original filtered back-projection (FBP) images. This model utilizes a U-Net–based convolutional neural network and was trained by receiving noise-added FBP images and producing original FBP-like images. DLIR was trained using images from different CT vendors (Siemens, Philips Healthcare, GE Healthcare, and Canon) with varying CT parameters (section thickness, mAs, kVp, contrast enhancement, convolution kernels, and automatic exposure control), so that it can work as a generalized vendor-agnostic denoising software [1]. Its training dataset was composed of more than one million CT images and 2100 different combinations of scan and reconstruction conditions; 80% of the images were used as the training dataset, and the other 20% comprised the validation dataset. This model can produce denoised images from original FBP images regardless of the reconstruction algorithm (e.g., soft or sharp kernel) and section thickness (e.g., 3 or 1 mm), and the clinical applicability of this model has been evaluated in several studies [2–5].

**REFERENCES**

1. Ahn CK, Heo C, Kim JH. Combined low-dose simulation and deep learning for CT denoising: application in ultra-low-dose chest CT. In: Fujita H, Lin F, Kim JH, editors. International Forum on Medical Imaging in Asia 2019. Singapore, Singapore: SPIE; 2019. p. 43.

2. Nam JG, Ahn C, Choi H, Hong W, Park J, Kim JH, et al. Image quality of ultralow-dose chest CT using deep learning techniques: potential superiority of vendor-agnostic post-processing over vendor-specific techniques. Eur Radiol. 2021;31:5139–47.

3. Hong JH, Park EA, Lee W, Ahn C, Kim JH. Incremental Image Noise Reduction in Coronary CT Angiography Using a Deep Learning-Based Technique with Iterative Reconstruction. Korean J Radiol. 2020;21:1165–77.

4. Kolb M, Storz C, Kim JH, Weiss J, Afat S, Nikolaou K, et al. Effect of a novel denoising technique on image quality and diagnostic accuracy in low-dose CT in patients with suspected appendicitis. Eur J Radiol. 2019;116:198–204.

5. Lim WH, Choi YH, Park JE, Cho YJ, Lee S, Cheon JE, et al. Application of Vendor-Neutral Iterative Reconstruction Technique to Pediatric Abdominal Computed Tomography. Korean J Radiol. 2019;20:1358–67.

**Supplementary Figures**

**Figure E1** Image noise and the signal-to-noise ratio were measured using region-of-interest in five different locations, including the lung parenchyma, trachea, aorta, muscle, and axillary fat. Image noise was defined as the standard deviation of the HU values within the area, while the signal-to-noise ratio was calculated from the absolute average HU value divided by the noise.


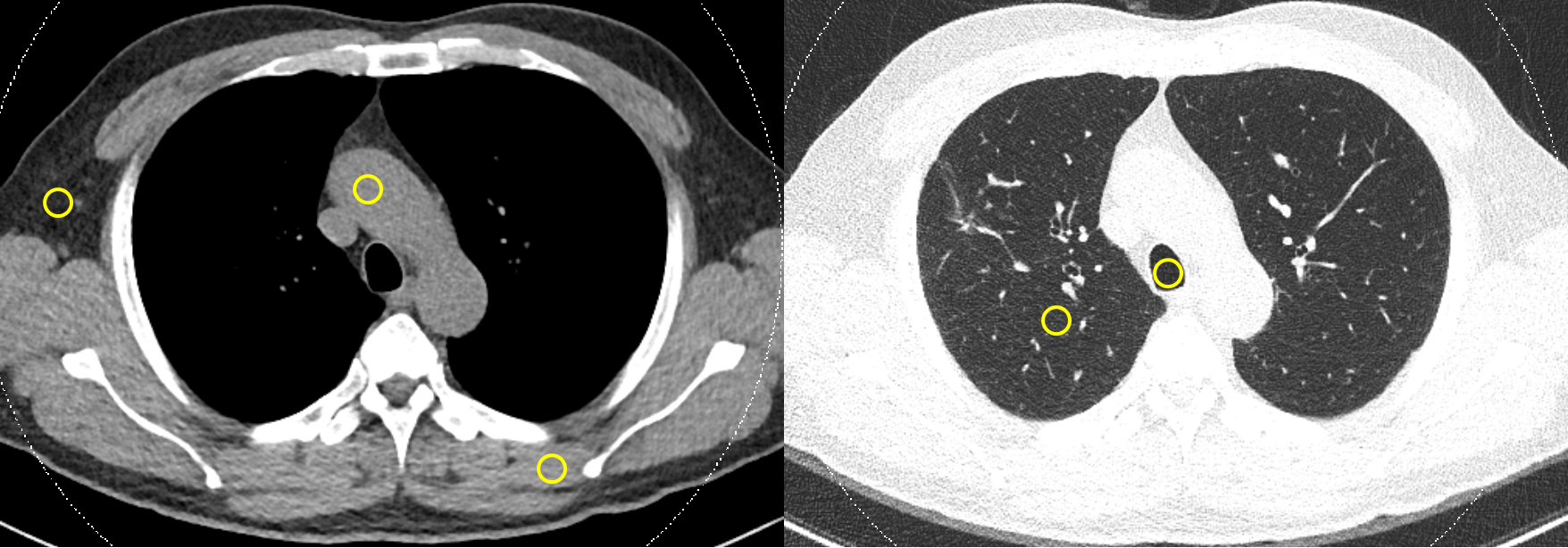


**Figure E2** Examples of beam-hardening artifacts. A woman received low-dose chest CT for the evaluation of thoracic metastasis. (A-B) Conventional low-dose chest CT images were reconstructed with iterative reconstruction, and (C-D) images generated using a quarter of the radiation were reconstructed with commercial deep-learning software. A higher level of beam-hardening artifacts was induced by a vertebral prosthesis on the quarter-dose images (arrowhead).

**
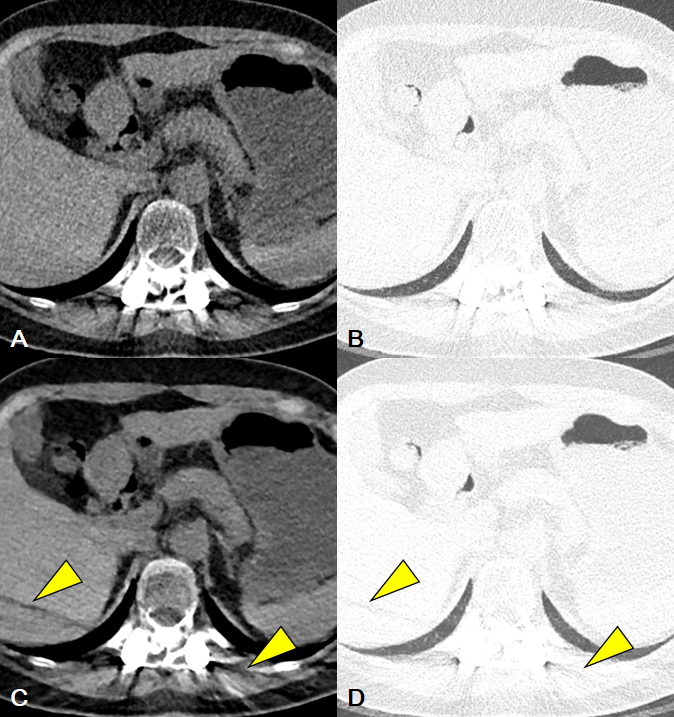
**

**Figure E3** An example of a false-negative nodule in lung nodule detectability assessment. A man received low-dose chest CT for the follow-up evaluation of underlying emphysema. A Lung-RADS category 3 nodule (mean diameter: 6.9 mm) was noted in the left lower lobe, and the nodule was clearly visualized on both (A, C) conventional low-dose chest CT images reconstructed using iterative reconstruction (LD-IR) and (B, D) images generated with a quarter of the radiation dose reconstructed using commercial deep-learning software (QLD-DLIR). One of the three thoracic radiologists correctly identified the nodule using both QLD-DLIR and LD-IR images, whereas another radiologist detected the nodule only when using LD-IR images, and the other radiologist failed to detect the nodule when using both QLD-DLIR and LD-IR images.

**
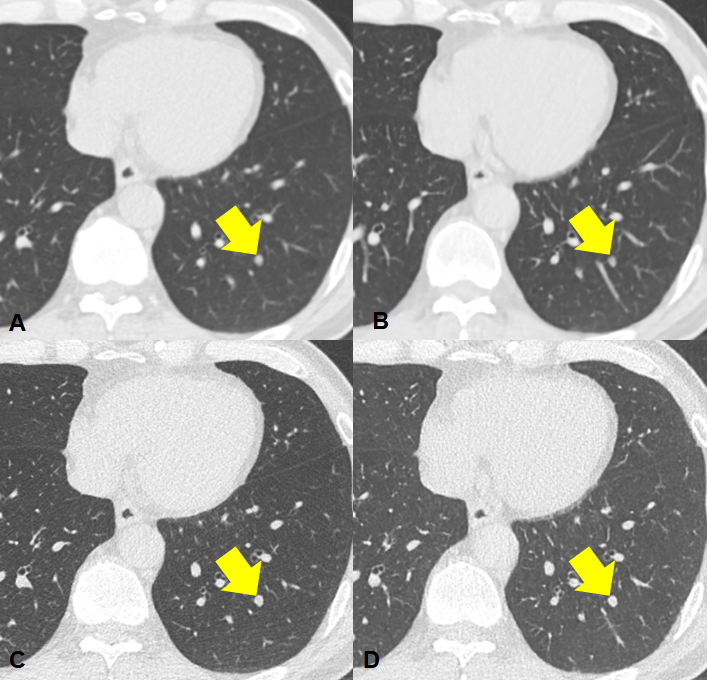
**

**Supplementary Tables**

**Table E1** Scoring Scale for the Qualitative Assessment of Image Quality

| **Parameter** | **Score** | **Scoring system** |
| --- | --- | --- |
| Subjective noise | 1–4 | 4: excellent image without noise; 3: some image noise, but no diagnostic difficulty; 2: moderate image noise with some diagnostic difficulty; 1: severe noise, nondiagnostic |
| Spatial resolution | 1–4 | For the lung-window setting,  4: all fissures are visualized with clear edge; 3: fissures can be identified, but with some blurring; 2: moderate blurring and invisible; 1: mostly invisible |
|  |  | For the mediastinal-window setting,  4: excellent, vessels/lymph nodes are clearly demarcated; 3: mostly visible, but some lymph nodes are difficult to delineate; 2: substantial difficulty in evaluating mediastinal lesions; 1: severely blurred, unable to distinguish structures |
| Distortion artifact | 1–4 | 4: not recognizable; 3: recognizable; 2: moderate to severe, but not affecting diagnostic performance; 1: severe, affecting diagnostic performance |
| Beam-hardening artifact | 1–4 | 4: not recognizable; 3: mild, adjacent anatomic structures could be observed and diagnosed; 2: moderate, the gross structure could still be observed, but the artifact affected the diagnosis; 1: severe, the bony structure and soft tissue near the artifact could not be observed and diagnosed. |
| Overall image quality | 1–4 | 4: excellent image quality; 3: good image quality without diagnostic limitation; 2: suboptimal, with some diagnostic limitation; 1: poor image quality, nondiagnostic |

**Table E2** Qualitative Image Quality Assessment Results on Supplementary 1-mm, Sharp-Kernel Images

|  | **QLD-DLIR** | **LD-IR** | ***P*-value*** |
| --- | --- | --- | --- |
| **Lung-window setting** |  |  |  |
| Subjective noise | 2.92 ± 0.25  (2.67, 3) | *3.12 ± 0.23*  *(3, 3.33)* | <.001 |
| Spatial resolution | 3.31 ± 0.30  (3, 3.33) | *3.80 ± 0.27*  *(3.67, 4)* | <.001 |
| Distortion artifact | 2.44 ± 0.20  (2.33, 2.67) | *2.71 ± 0.15*  *(2.67, 2.67)* | <.001 |
| **Mediastinal-window setting** |  |  |  |
| Subjective noise | *2.04 ± 0.19*  *(2, 2)* | 1.93 ± 0.20  (1.67, 2) | <.001 |
| Spatial resolution | 2.73 ± 0.26  (2.67, 3) | *2.87 ± 0.33*  *(2.67, 3)* | .001 |
| Distortion artifact | 2.47 ± 0.23  (2.33, 2.67) | *2.69 ± 0.23*  *(2.67, 3)* | <.001 |
| **Beam-hardening artifact** | 2.99 ± 0.35  (2.75, 3.33) | *3.39 ± 0.27*  *(3.33, 3.67)* | <.001 |
| **Overall image quality** | 2.73 ± 0.32  (2.67, 3) | *3.12 ± 0.26*  *(3, 3.33)* | <.001 |

Note.—Data are presented as means ± standard deviations (interquartile range). Higher scores indicate better image quality. Italicized data indicate that the values are higher than the compared counterpart. DLIR=deep-learning image reconstruction, IR=iterative reconstruction, LD=low dose, QLD=quarter of the low dose.

* *P*-values were calculated using the paired *t*-test or Wilcoxon signed-rank test, as appropriate.

**Table E3** Patient-Based Estimates of the Detection of Lung-RADS Category 3 or 4 Nodules

|  | Sensitivity |  |  | Specificity |  |  |
| --- | --- | --- | --- | --- | --- | --- |
|  | QLD-DLIR | LD-IR | *P*-value* | QLD-DLIR | LD-IR | *P*-value* |
| Reader 1 | 73.3%  (22/30) | 76.7%  (23/30) | 1.00 | 80.0%  (56/70) | 82.9%  (58/70) | .75 |
| Reader 2 | 83.3%  (25/30) | 80.0%  (24/30) | 1.00 | 95.7%  (67/70) | 94.3%  (66/70) | 1.00 |
| Reader 3 | 80.0%  (24/30) | 86.7%  (26/30) | .50 | 74.3%  (52/70) | 75.7%  (53/70) | 1.00 |
| Pooled readers | 78.9%  (71/90) | 81.1%  (73/90) | .45 | 83.3%  (175/210) | 84.3%  (177/210) | .74 |

Note.—DLIR=deep-learning image reconstruction, IR=iterative reconstruction, LD=low dose, Lung-RADS=lung imaging reporting and data system, QLD=quarter of the low dose.

**P*-values were calculated from either McNemar test for individual radiologists or generalized estimating equations for pooled radiologists.
